# Transcriptomic buffering detection: a computational approach to identifying essential gene dependencies in pancreatic cancer

**DOI:** 10.1093/bioadv/vbag280

**Published:** 2026-10-07

**Authors:** Magnus Boman, Eleni Afentaki, Julia Marie Greely, Noura Ezaz-Nikpay, Rula Zain, C I Edvard Smith

**Affiliations:** Division of Clinical Epidemiology, Department of Medicine Solna, Karolinska Institutet, SE-171 76 Stockholm, Sweden; Department of Laboratory Medicine, Karolinska Institutet, SE-141 83 Stockholm, Sweden; Shiley-Marcos School of Engineering, University of San Diego, San Diego, CA, 92110, United States; Department of Computing, Imperial College, London, SW7 2AZ, United Kingdom; Department of Laboratory Medicine, Karolinska Institutet, SE-141 83 Stockholm, Sweden; Karolinska ATMP Center, Karolinska Institutet, SE-171 64 Stockholm, Sweden; Department of Clinical Genetics and Genomics, Centre for Rare Diseases, Karolinska University Hospital, SE-141 86 Stockholm, Sweden; Department of Laboratory Medicine, Karolinska Institutet, SE-141 83 Stockholm, Sweden; Karolinska ATMP Center, Karolinska Institutet, SE-171 64 Stockholm, Sweden

## Abstract

**Motivation:**

Loss-of-function (LOF) mutations in essential genes (Goners) should be lethal, yet some tumours survive through compensatory buffering mechanisms. Existing computational methods for identifying synthetic lethal (SL) interactions focus on known tumour suppressor losses, missing essential gene dependencies. We developed a computational pipeline to identify SL buffering mechanisms by correlating Goner LOF burden with transcriptomic compensation.

**Results:**

Applying our pipeline to pancreatic adenocarcinoma (PAAD), we integrated CRISPR essentiality screens (DepMap), mutation data, and RNA expression profiles from 165 TCGA-PAAD cases. This PAAD analysis supports the feasibility of the pipeline but does not nominate a transcriptomic buffer; applicability to other cancer types remains to be tested.

**Availability and implementation:**

Analysis code and documentation are available at https://github.com/d3dd/goblet. Implemented in Python 3.9+ using pandas, scipy, and gseapy. All data are publicly available from TCGA and DepMap.

## 1 Introduction

Cancer cells acquire mutations that drive proliferation, but not all mutations confer selective advantage. We previously identified a class of mutations termed ‘Goners’, genetic alterations whose presence imposes fitness costs, reflected by their absence or low variant allele frequencies in viable tumour populations (Bunz 2024, Smith and Zain 2024). These mutations represent an under-explored therapeutic vulnerability: if tumour cells harbouring such alterations survive, they must employ compensatory mechanisms to mitigate the toxic effects.

Synthetic lethality (SL) describes genetic interactions where simultaneous disruption of two genes is lethal, whilst loss of either alone is tolerated (Dobzhansky 1946). In cancer therapeutics, SL approaches traditionally exploit tumour suppressor gene (TSG) loss: when a tumour loses gene A (e.g. *BRCA1/2*), it becomes dependent on gene B (e.g. *PARP1*), creating a therapeutic target. Recent work by Haider *et al*. (2025) developed SYLVER, a computational pipeline that integrates DepMap CRISPR–Cas9 screen data with transcriptomic profiles from 9316 TCGA tumours across 32 cancer types, demonstrating that transcriptional buffering of TSG loss by hyperexpression of SL partner genes is a common, clinically observable phenomenon. However, SYLVER and existing computational methods focus exclusively on TSG loss, genes whose absence is tolerated or selected for in cancer. Essential genes whose loss-of-function (LOF) would normally be lethal represent a distinct and harder problem: when such ‘Goner’ LOF mutations occur yet tumours survive, compensatory buffering may be present, but the rarity of individual Goner LOF events means single-gene correlation approaches may lack power, motivating a burden-based aggregation strategy. This depletion-based logic has precedent in germline constraint analysis (Karczewski *et al*. 2020).

We developed a computational pipeline named GOBLET (Goner-Orchestrated Buffering via Lethality and Expression in Tumours) to explore SL-buffering mechanisms associated with essential gene loss. The approach integrates CRISPR-based essentiality screens (DepMap), somatic mutation data, RNA expression profiles (TCGA), and curated SL interaction databases. Because individual Goner LOF events are rare by definition, as cells acquiring them tend to be eliminated, no single Goner gene accumulates sufficient LOF events to power gene-level correlation. We therefore aggregate across all common-essential genes to compute a per-sample Goner LOF burden score, then correlate this burden with genome-wide expression to identify genes whose upregulation co-varies with essential gene dysfunction. Filtering for established SL partnerships (SynLethDB) subsequently prioritizes candidates with a mechanistic prior for buffering. Unlike SYLVER, which correlates individual TSG defects with SL partner expression, GOBLET’s burden-based aggregation is designed specifically for the low-frequency Goner alteration class.

A systematic review of the literature identified 24 articles relevant to pan-cancer Goner gene identification from an initial set of 2596 hits (Fig. 1, available as supplementary data at *Bioinformatics Advances* online and Tables 1 and 2, available as supplementary data at *Bioinformatics Advances* online). The majority (59%) employed CRISPR-based screening methods. Notably, a benchmarking study of 12 supervised deep learning models for SL pair classification found that performance degrades substantially when neither gene of a tested pair appears in the training set (Feng *et al*. 2024), motivating our correlation-based approach which requires no prior SL labels for the Goner alteration class.

**Figure 1:**
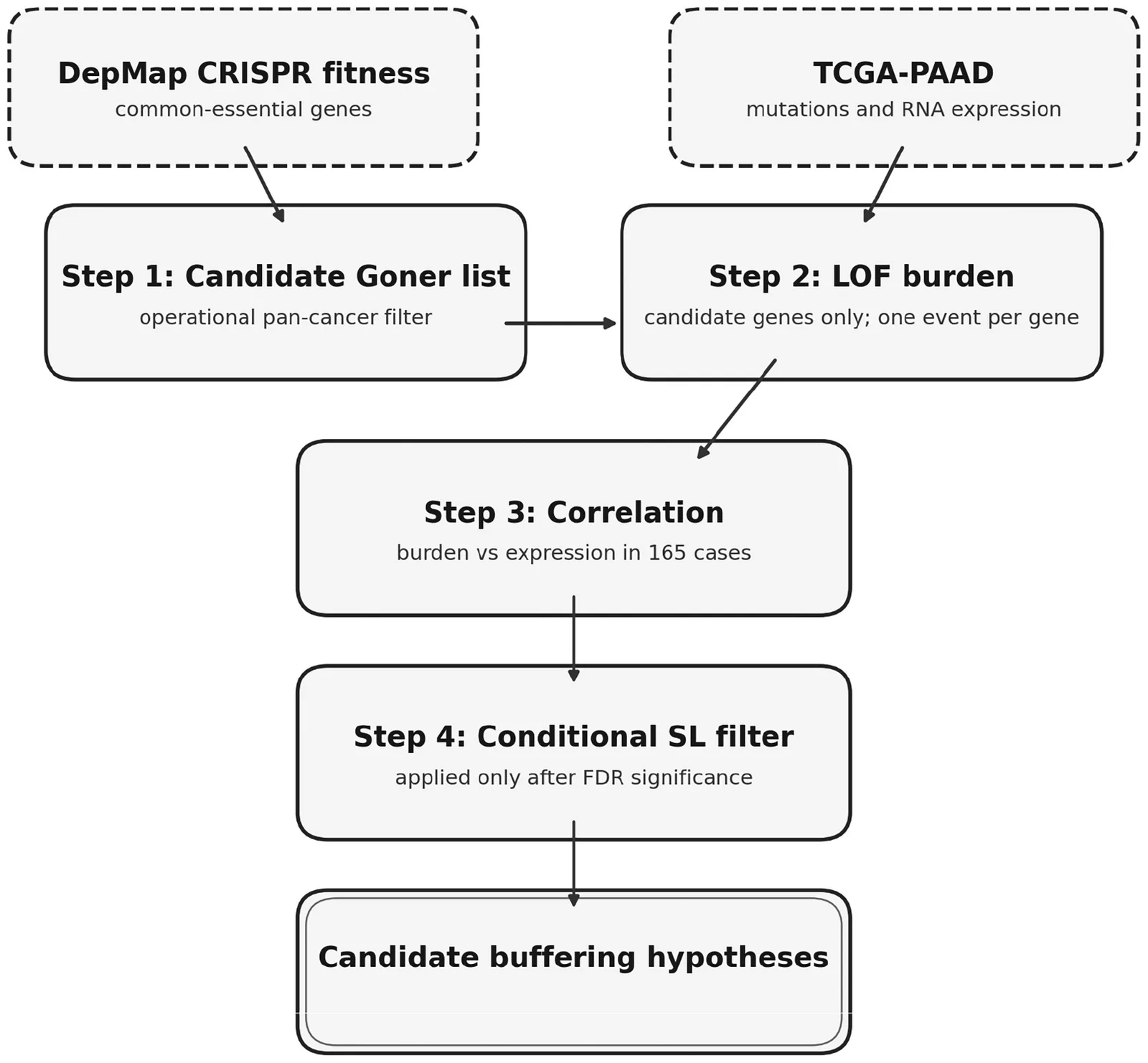
Overview of the GOBLET computational pipeline. Dashed boxes indicate input data sources; solid boxes indicate pipeline steps; the double-bordered box indicates the final output. Step 1 derives the Goner list from DepMap 23Q2 CRISPR fitness scores (Chronos <−0.5 in ≥90% of cell lines). Step 2 computes a per-case candidate-only LOF burden score from TCGA-PAAD somatic mutation data. Step 3 correlates LOF burden with genome-wide RNA-seq expression across 165 TCGA-PAAD cases (Spearman ρ). Step 4 filters positively correlated genes for established synthetic lethal partnerships (SynLethDB) if an expression association passes multiple-testing correction.

We demonstrate proof-of-concept in pancreatic adenocarcinoma (PAAD), a cancer with poor prognosis and limited therapeutic options. We also studied enrichment of branched-chain amino acid (BCAA) catabolism as an exploratory downstream analysis, because this metabolic pathway has established roles in PAAD progression (Mayers *et al*. 2014, Ericksen *et al*. 2019, Zhu *et al*. 2020). This comparison against known biology illustrates how statistically supported GOBLET outputs could be assessed for biological relevance.

## 2 Methods

Figure 1 outlines the four-step GOBLET pipeline; each step is described in detail below.

### 2.1 Data

We leverage multiple open-access resources to implement a buffering detection pipeline. First, the DepMap Cancer Dependency Map provides genome-wide CRISPR-Cas9 gene-effect scores (e.g. CERES/Chronos) and RNA-seq expression with accompanying metadata across hundreds of cancer cell lines. We use these data to identify an operational set of candidate Goners, as genes with strong negative essentiality scores (e.g. score less than −0.5 in at least 90% of lines), and to explore context-specific buffering signals. Second, The Cancer Genome Atlas (TCGA) offers comprehensive tumour datasets including somatic mutations, transcript levels (RNA-seq), and clinical outcomes for >10 000 patients. We collate TCGA-PAAD mutation and RNA-expression matrices by case identifier, yielding 165 cases, to compare candidate Goner LOF burden with expression profiles. Curated SL interaction databases (e.g. SynLethDB, SL-Miner) are used to filter candidate buffer genes that co-function with Goners after an expression association passes the statistical screen. To facilitate modelling and reproducibility, we also plan to use publicly available integrated data resources, such as TCGA–DepMap hybrid datasets, which link cell-line dependency profiles with tumour expression and mutation patterns. These resources could enable a unified multi-omics approach to detect transcriptomic buffering and prioritise clinically relevant SL targets.

### 2.2 Hypothesis-driven strategy

Goners can be brought about by genetic or epigenetic gain of function (GOF) (Bunz 2024, Smith and Zain 2024) or LOF (Smith *et al*. 2024) changes. Our hypothesis predicates that any tumour cell, irrespective of whether it acquires lethal GOF or LOF alterations, must be buffered for continued survival. In this study, we focus on the LOF scenario in which a cell obtaining a lethal mutation survives thanks to compensatory buffering mechanisms that existed prior to the occurrence of the LOF. Typically, for Goners belonging to the group of LOF mutations, the underlying mechanism is the upregulation of an SL partner gene.

We posit that only a restricted subset of genes possesses the regulatory and functional properties necessary to act as effective transcriptional buffers upon Goner gene perturbation. Genes not endogenously expressed in the tissue of origin are unlikely to be transcriptionally induced without substantial epigenetic reprogramming. Even in cases where rare subclonal populations upregulate such genes, the lack of functional compensation or the imposition of a metabolic burden may mitigate any selective advantage. Consequently, buffering is likely constrained to genes that are: (i) constitutively expressed in the tissue, (ii) non-essential under basal conditions, and (iii) capable of substituting key network functions lost through SL.

We identify candidate Goner mutations using DepMap and non-isogenic CRISPR screens. We assume that transcriptomic compensation will manifest as consistent overexpression of SL partners in tumours that tolerate Goner loss. DepMap scores are thus treated as proxy for lethality, and we assume buffering can be inferred from overexpression. We excluded genes that were not essential in >90% of cancer cell lines, a conservative heuristic that we can vary as necessary in sensitivity analyses of our model.

Non-isogenic screens such as DepMap profile diverse cancer cell lines, capturing context-dependent essentiality but introducing potential confounders from heterogeneous genetic backgrounds. Notably, Haider *et al*. (2025) demonstrate that non-isogenic DepMap SL signals partially overlap with gold-standard isogenic screen results, and that this overlap strengthens when combined with expression buffering, validating the approach used here.

### 2.3 Prediction model

To identify and evaluate candidate buffering relationships for Goner genes, we first interrogate TCGA to detect tumour samples with predicted LOF mutations in putative Goner genes, identified from DepMap non-isogenic CRISPR screens as those with Chronos/CERES scores <−0.5 in at least 90% of cancer cell lines. We acknowledge that essentiality scores may vary across tumour types; however, we use this pan-cancer threshold as an operational filter for broadly essential genes. We use the 90% threshold as a conservative proxy for ‘common-essential’ genes; although no universally accepted cut-off exists, DepMap and related literature frequently apply a percentile-based criterion to define broadly essential genes. For each case, the LOF burden was the number of distinct candidate genes with at least one predicted LOF variant; multiple variants in the same gene and case were counted once, and genes outside the candidate set did not contribute to the score.

We correlated candidate-only LOF burden with genome-wide RNA expression using Spearman’s rank correlation. Multiple-testing correction was implemented using the Benjamini–Hochberg procedure, with a positive association requiring ρ>0.3 and adjusted P<.05. The test was repeated after excluding the case with the largest mutation and candidate-LOF burdens. Curated SL relationships were reserved for subsequent filtering of statistically supported associations. Expression normalisation and filtering are described in the Supplementary Methods, available as supplementary data at *Bioinformatics Advances* online.

We selected PAAD for proof-of-concept given its poor prognosis (5-year survival <10%), limited therapeutic options, and well-characterised genomic landscape, with predominating *KRAS* mutations, in TCGA. To identify tumours with predicted Goner loss, we used the TCGA MAF file to parse mutation calls for LOF variants in the candidate set. We are aware that Goner gene gain-of-function (GOF) mutations could play a similar role, but we leave that for future research. Next, in the RNA expression comparison step, we use the TCGA RNA-seq matrix to compare gene expression profiles according to candidate-only LOF burden. We focus on identifying genes positively correlated with the burden. Then we filter the list of positively correlated genes to retain only the SL partners of the lost Goner(s), retaining pairs only where the expression association is statistically supported. The disease types for PAAD included adenomas and adenocarcinomas; cystic, mucinous, and serous neoplasms; ductal and lobular neoplasms; epithelial neoplasms, not otherwise specified.

## 3 Results

After parsing the DepMap gene dependency summary, filtering for Chronos dataset and common-essential genes, we identified 1526 candidate Goners. We set up a data frame in Python and added filtered LOF entries: 1872 out of 24 849 total rows obtained from TCGA. We next aligned 165 cases between LOF and RNA data. From 1872 LOF entries, we got 1599 unique genes. Restricting the burden to the candidate set yielded 126 candidate-gene/case LOF observations, of which 93 occurred in one hypermutated case containing 17 551 somatic variant records, compared with a cohort median of 39.

No gene met both ρ>0.3 and adjusted P<.05. The largest positive association was for *PARD6B* (ρ=0.342, nominal $P=6.95\times 10^{-6}$, adjusted P=.121). Excluding the hypermutated case did not produce an False Discovery Rate (FDR)-significant result; the smallest adjusted *P* was 0.101, again for *PARD6B*.

Given the biological relevance of amino acid catabolism in PAAD, we further examined whether additional enzymes involved in BCAA metabolism might show correlated expression patterns. To assess the specificity of the BCAA signal, we examined three additional enzymes known to regulate BCAA degradation: BCKDK, DLD, and PPM1K. No expression association passed the candidate-only FDR screen; these analyses therefore do not establish BCAA-related buffering in this cohort.

## 4 Conclusion

### 4.1 Biological significance and therapeutic implications

PAAD is a homogeneous disease from a mutation point of view, since more than 90% of the tumours carry mutations in the *KRAS* gene (Waddell *et al*. 2015). This is advantageous when searching for Goners, because Goner types are expected to vary according to the pathways altered by Drivers. In this TCGA-PAAD analysis, no association passed the specified false-discovery threshold, and no transcriptomic buffer or therapeutic dependency was nominated.

Although the present analysis cannot nominate individual buffers, many of the genes examined as putative buffering candidates in the exploratory downstream analysis appear to be involved in cellular metabolism. Notably, genes such as FPGS, PRKAR1A, AOC1, AADAC, ABCA3, GOT2, DPYSL3, FKBP3, ACY1, ACAT1, and GCK are all known or predicted to play roles in metabolic pathways. Other genes in this exploratory set suggest mechanistic diversity. LMNA and NUP155 are both associated with nuclear architecture and transport. BNIP2 encodes a BCL2-interacting protein involved in apoptosis regulation, a key process in tumour progression. POLE encodes DNA polymerase epsilon, with essential roles in DNA replication and repair. GRK6 is a G-protein-coupled receptor kinase with potential relevance to signal transduction. Immune and inflammatory markers include CFH (Complement Factor H), implicated in immune modulation and age-related macular degeneration, and CRP, a well-established inflammatory biomarker often increased in various cancers, including pancreatic cancer. GATA3 is a transcription factor involved in lineage specification. PRPF31 encodes a splicing factor with high expression in pancreatic tissue. CDC25C, a cell cycle regulator, has been proposed as a prognostic marker in pancreatic cancer. KMT2D, a chromatin remodeller, and LCK, a tyrosine kinase typically associated with lymphocytes, also featured amongst the examined genes. Together, these functional annotations illustrate that a putative buffering set may be mechanistically diverse, potentially involving metabolism, transcriptional regulation, nuclear organisation, and cell cycle control.

Although our exploratory enrichment analysis cannot be interpreted as evidence of a BCAA buffering mechanism, BCAA metabolism remains biologically relevant in PAAD. The results from Mayers *et al*. (2014) suggest that elevation of circulating BCAA is an early event in human PAAD development. Moreover, tumour-reprogrammed stromal BCAT1 can fuel branched-chain ketoacid dependency in stromal-rich PDAC tumours (Zhu *et al*. 2020). For example, knockdown of BCAT2 or BCKDHA, key enzymes in BCAA breakdown, both significantly impair proliferation of pancreatic ductal adenocarcinoma cells, but not of non-malignant pancreatic cells (Ericksen *et al*. 2019). These observations motivate future testing of whether statistically supported GOBLET signals identify metabolic buffering dependencies.

Additionally, impaired BCAA catabolism has been observed in hepatocellular carcinomas and is predictive of poor clinical outcomes (Ericksen *et al*. 2019). Targeting BCAA catabolic pathways may therefore offer a dual inhibition strategy: it could impair cancer growth and, if future work establishes a role in buffering, disrupt tumour metabolic compensation and thereby reveal SL vulnerabilities. Such an approach is consistent with emerging research leveraging metabolic dependencies in cancer for therapeutic gain.

It is plausible that only a restricted subset of genes possesses the regulatory and functional properties necessary to act as effective transcriptional buffers upon Goner gene perturbation. Genes not endogenously expressed in the tissue of origin are unlikely to be transcriptionally induced without substantial epigenetic or transcriptional reprogramming. Even in cases where rare subclonal populations upregulate such genes, the lack of functional compensation or the imposition of a metabolic burden may mitigate any selective advantage. Consequently, buffering is likely constrained to genes constitutively expressed, non-essential under basal conditions, and capable of substituting key network functions lost through SL. Clonal heterogeneity may give rise to variable growth rates; however, only those clones in which buffering confers a net fitness benefit are expected to persist. This selective bottleneck may underlie the recurrent identification of specific buffering candidates across tumour samples. For genes whose LOF has broad downstream regulatory consequences, such as genes involved in epigenetic silencing, where LOF leads to genome-wide de-repression, the observed positive correlation may reflect indirect transcriptional effects rather than directed compensatory buffering, and such cases cannot be distinguished by the pipeline without additional functional validation. The pipeline therefore performs best when applied to Goner candidates whose primary function is not transcriptional regulation.

### 4.2 Limitations and future directions

The pipeline identifies correlation between Goner LOF burden and gene expression—consistent with transcriptional buffering but not proof of causation. A null result from this cross-sectional burden–expression test does not exclude alteration-specific or non-transcriptional buffering, which may fall outside the tested signal or be obscured by sparse and uneven burden, hypermutation, or other confounding factors.

The pan-cancer essentiality threshold (Chronos <−0.5 in ≥90% of DepMap cell lines) may not reflect PAAD-specific dependencies. Future extensions should examine negatively correlated genes, generalise to additional cancer types with subtype stratification (e.g. breast cancer), and incorporate tumour-type-specific essentiality thresholds. They should also account for total mutation burden, driver context, tumour purity, and copy number, and be validated in independent cohorts.

## Supplementary Material

vbag280_Supplementary_Data

## Data Availability

The complete analysis pipeline is implemented in Python 3.9+ and available at https://github.com/d3dd/goblet under MIT licence. The pipeline requires pandas (≥1.3.0), scipy (≥1.7.0), numpy (≥1.21.0), and gseapy (≥1.0.0). All input data are publicly available: TCGA-PAAD mutation and expression data from GDC Data Portal, DepMap CRISPR dependency scores from DepMap Portal (release 23Q4), and curated synthetic lethal pairs from SynLethDB. Documentation includes instructions for reproducing the PAAD analysis.
