# Supplementary material for: Transcriptomic buffering detection: a computational approach to identifying essential gene dependencies in pancreatic cancer
Source: Bioinform Adv. 2026 Oct 7;6(1):vbag280. doi: 10.1093/bioadv/vbag280 (PMC13644534; doi:10.1093/bioadv/vbag280)
Supplement: Supplementary file 1 [file vbag280_supplementary_data.pdf]

Supplementary Material for: Transcriptomic Buffering Detection: A Computational Approach to Identifying Essential Gene Dependencies in Pancreatic Cancer

Magnus Boman, Eleni Afentaki, Julia Marie Greely, Noura Ezaz-Nikpay,
Rula Zain and C.I. Edvard Smith

Supplementary Figure **(see next page)**

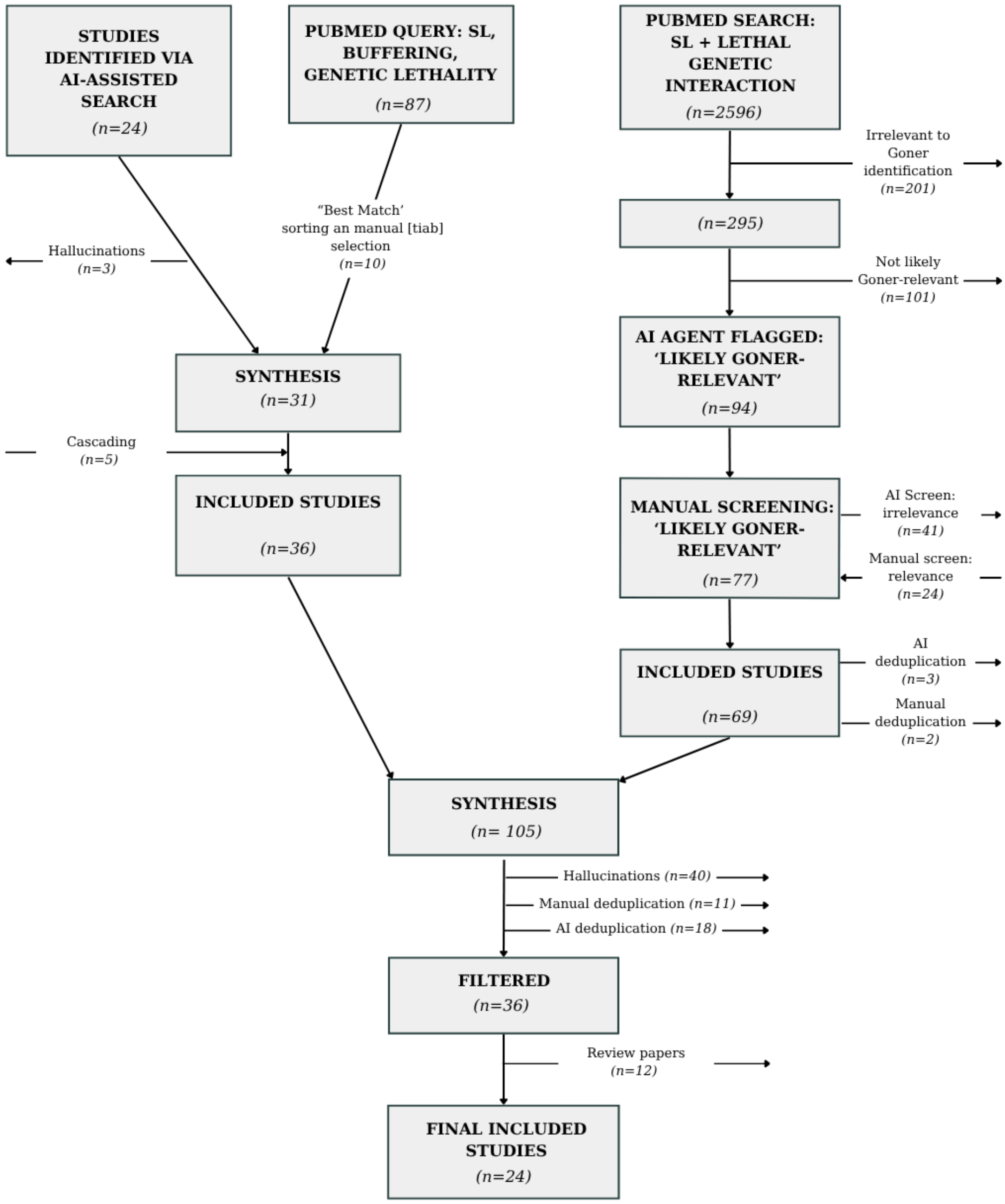

Figure 1: **Supplementary Figure S1. PRISMA-ScR flow diagram for scoping review synthesis.** Syncing two individual scoping reviews, with one (at right) casting a wide net over four databases, and the other (at left) focusing on PubMed. The resulting PRISMA-ScR funnelled 24 articles to be analysed further. Exclusion criteria: non-English and publications before 2015. AI model used was GPT4o, all answers of which were manually double-checked by at least two co-authors. In the final step, review papers were excluded to leave only original research, with the reviews being analysed separately.

Supplementary Tables

Scoping Review Results

Casting a wide net, we first identified articles relevant to Goner gene identification, funnelled down from 2,596 hits to 69 (Supplementary Table S1). The majority (59%) of these 69 articles employed CRISPR-based screening methods, including genome-wide dropout and essentiality profiling. An additional 38% were experimental studies using viability assays or dual-gene inhibition without genome-wide screening. Only one computational and one CRISPR-Cas9-specific study remained post-deduplication. The most common cancer context was pan-cancer ($n = 33$), followed by breast ($n = 9$), lung ($n = 5$), glioblastoma ($n = 4$), colorectal ($n = 4$), and prostate ($n = 3$), with remaining studies spanning leukaemia, ovarian, gastric, and hepatocellular carcinomas. The predominant model system was cancer cell lines ($n = 17$), with additional use of organoids, tumour biopsies, and TCGA-derived data. Goner gene identification methods were highly heterogeneous, but most centred on CRISPR dropout, fitness profiling, or gene essentiality scoring. Validation approaches primarily included growth inhibition, viability assays, and dropout reproducibility analysis. These 69 studies could, in theory, provide a structured foundation for further candidate gene prioritisation, but as they were scrutinised in view of a second scoping review, independently conducted by another researcher, almost all out of the 69 articles lacked relevance.

Category	Summary
**Study types**	CRISPR screen (41), Experimental (26), CRISPR-Cas9 screen (1), Computational (1)
**Cancer types**	Pan-cancer (33), Breast (9), Lung (5), Glioblastoma (4), Colorectal (4), Prostate (3), Leukaemia (3), Ovarian (2), Gastric (1), Liver (1), Hepatocellular carcinoma (1), MSI-high (1), Various/unspecified (2)
**Model systems**	Cancer cell lines (17), Cell lines (16), Lung cancer cells (2), Leukaemia models (2), Breast tumour biopsies (2), Organoids (1), Glioblastoma models (1), TCGA data (1), Prostate models (1), Others/Unspecified (26)
**Goner ID methods**	CRISPR dropout screens, essentiality profiling, dual inhibition assays, synthetic lethal fitness assays
**Validation methods**	Fitness profiling, viability assays, dropout scoring, essential gene scoring, growth inhibition

Table 1: **Supplementary Table S1. Summary of initial scoping review ($n = 69$ articles).** Types of deduplicated Goner-relevant papers identified in the first scoping review across four databases.

In a second and independent scoping, a total of 36 separate articles were identified as relevant to Goner gene identification (Supplementary Table S2). Of these articles, 31% employed CRISPR-based screening methods and 28% employed computational or machine learning approaches. Additionally, 25% of the articles were lab-based experimental studies focusing on validation utilising viability assays, genetic knockouts, and drug response profiling. The remaining 17% consisted of review articles with topics surrounding synthetic lethality identification and methods.

Category	Summary
**Study types**	CRISPR (11), Computational/ML (10), Experimental validation (9), Review (6)
**Cancer types**	Pan-cancer (15), Breast (5), Colorectal (4), Lung (3), Glioblastoma (2), Other (7)
**Model systems**	Cancer cell lines (majority), Organoids, TCGA data, Patient-derived models
**Key methods**	CRISPR screens, ML prediction models, Transcriptomic analysis, Network analysis

Table 2: **Supplementary Table S2. Summary of second independent scoping review (*n* = 36 articles).** Distribution of study types and cancer contexts in the independently conducted second scoping review.

Supplementary Methods

Detailed Scoping Review Methodology

The scoping review followed PRISMA-ScR (Preferred Reporting Items for Systematic Reviews and Meta-Analyses extension for Scoping Reviews) guidelines. Two independent reviewers conducted parallel searches across four databases: PubMed, Web of Science, Scopus, and IEEE Xplore. Search terms included combinations of "Goner", "essential gene", "synthetic lethality", "genetic buffering", "CRISPR screen", and related terms. Inclusion criteria required: (1) original research articles, (2) English language, (3) publication date 2015 or later, (4) focus on essential gene identification or synthetic lethality in cancer. Exclusion criteria included: conference abstracts, non-peer-reviewed preprints, and studies not relevant to cancer biology.

The first scoping review identified 2,596 initial hits, which were screened by title and abstract to yield 69 potentially relevant articles. The second independent scoping review focused on PubMed and identified 36 relevant articles. Both sets were merged and deduplicated, with conflicts resolved through discussion between reviewers. The final synthesis included 24 original research articles deemed directly relevant to Goner gene identification and synthetic lethality detection. An AI model (GPT-4o) was used to assist with initial screening, but all decisions were manually verified by at least two co-authors.

Statistical Analysis Details

Spearman Correlation. For each of 165 aligned TCGA-PAAD cases, candidate-only LOF burden was defined as the number of distinct genes in the operational candidate set carrying at least one predicted LOF variant. Genes outside the candidate set did not contribute to the score. We computed Spearman's rank correlation coefficient (ρ) between gene expression levels and this burden. Raw expression counts were converted to counts per million and transformed as $\log_2(\text{CPM}+1)$; genes expressed at ≥ 1 CPM in at least 20% of cases were tested. The test was also performed after excluding the case with the largest mutation and candidate-LOF burdens.

Multiple Testing Correction. We applied the Benjamini–Hochberg false discovery rate (FDR) correction across all tested genes. A positive association required $\rho > 0.3$ and adjusted $p < 0.05$.

Downstream Analysis. Curated synthetic-lethal filtering and gene-ontology analysis were to be applied only if an expression association passed the statistical screen.

Computational Environment

All analyses were performed in Python 3.9.7 with the following package versions:

- pandas 1.3.5
- numpy 1.21.6
- scipy 1.7.3
- gseapy 1.0.4
- matplotlib 3.5.1
- seaborn 0.11.2

Computing was performed on a standard desktop workstation (Intel Core i7-10700K, 32GB RAM, Ubuntu 22.04 LTS).
